# The psychometric properties of the Barkin index of maternal functioning (BIMF) for the Iranian population

**DOI:** 10.1186/s12905-019-0859-2

**Published:** 2019-12-21

**Authors:** Mojgan Mirghafourvand, Jennifer L. Barkin, Mohammad Asghari Jafarabadi, Fatemeh Karami, Solmaz Ghanbari-Homayi

**Affiliations:** 1 Social determinants of Health Research Center, Tabriz University of Medical Sciences, Tabriz, Iran; 2 Department of Community Medicine, Mercer University School of Medicine, Macon, GA USA; 3 Department of Statistics and Epidemiology, Tabriz University of Medical Sciences, Tabriz, Iran; 4 Road Traffic lnjury Research Center, Tabriz University of Medical Sciences, Tabriz, Iran; 5 MSc Student of Midwifery, Students’ Research Committee, Tabriz University of Medical Sciences, Tabriz, Iran; 6 Department of Midwifery, Faculty of Nursing and Midwifery, Tabriz University of Medical Sciences, Tabriz, Iran

**Keywords:** Motherhood function, Postpartum period, Validity, Reliability, Psychometric, Iran

## Abstract

**Background:**

Mothers’ capability for childcare and compatibility with the maternal role represent important challenges in postpartum care. Given the significance of evaluating maternal functioning, and the lack of adequate standard instruments in Iran for this purpose, the present study was aimed at translating and conducting a psychometric assessment of the Barkin Index of Maternal Functioning (BIMF) for Iranian women.

**Methods:**

The instrument was translated into Persian using the Backward Forward method. The study included 530 women in the postpartum period admitted to healthcare centers in Tabriz, Iran; they were selected through the cluster sampling method. Face, content, and construct (through exploratory and confirmatory analyses) validity were presently examined. Reliability of the questionnaire was determined using the internal consistency and test-retest reliability methods.

**Results:**

Two factors (mom’s needs and competency), emerged based on exploratory factor analysis. The *x*^*2*^*/df* ratio was less than 5, and the values of the Root Mean Square Error of Approximation (RMSEA) and the Root Mean Square Residual (RMR) were less than 0.08 and 0.1, respectively, verifying the model validity. Cronbach’s alpha coefficient and Intra-class Correlation Coefficient (ICC) were calculated as 0.88 and 0.85, respectively, indicating reliability.

**Conclusion:**

The Persian version of the BIMF is a valid and reliable instrument for measuring the postpartum functioning of Iranian mothers.

## Background

Childbirth is a significant and life-altering experience for women who choose to have children [1]. In the postpartum period, women face significant changes in their cognitive, behavioral, and social functions [2, 3] and are required to adapt and integrate additional responsibilities into their existing set of roles and responsibilities [4]. This process can be both enjoyable and extremely challenging [5].

Postpartum maternal functioning encompasses various dimensions, including personal care, child and family care, and social and occupational activities [6]. Maternal functioning is reportedly improved substantially between the first and sixth postpartum weeks [7]; however, a number of women take up to six months to achieve a desirable level of functioning [8].

Accordingly, levels of maternal functioning vary among mothers, with numerous contributing factors such as maternal age, education, parity, unintended pregnancy, type of delivery [9], postpartum maternal mental health status [9], perceived support [10], and the prevailing sociocultural perspectives in society [11]. Optimal maternal functioning contributes positively to neonatal development and maternal-neonatal bonding, whereas suboptimal functioning may operate in an opposite fashion [11, 12].

Proper assessment of postpartum maternal functioning is key to identifying mothers who might be struggling in the maternal role and require extra support in order to adapt optimally. In a recent study conducted in the United States, BIMF scores were 16 points higher (*p* < 0.0001) on average, after women participated in a community-based non-clinical intervention [13]. The intervention consisted of trained volunteers providing social support to new mothers who either requested it or were referred. This results of this study point to the powerful effect of social support in the postpartum period.

Until recently, the Inventory of Functional Status After Childbirth (IFSAC) was the only instrument developed to measure postpartum maternal functioning. However, subsequent reports have pointed to potential dubious research results in studies using unmodified versions of this instrument. This is because the IFSAC was developed based on the assumption that optimal postpartum maternal functioning is contingent upon mothers resuming the majority of their prepartum responsibilities [14–16]. However, prepartum and postpartum functioning are not comparable, as childbirth often requires a reevaluation of one’s activities. In fact, due to the IFSAC’s scoring algorithm and inherent penalty for reprioritization after childbirth, the majority of new mothers found it difficult to return to their previous functional status [16, 17]. Additionally, maternal psychological well-being, women’s understanding of their new role, and their thoughts and feelings about adjustment over the first postpartum year, are not factored into the IFSAC’s assessment of functioning [6].

The BIMF was developed as a multidimensional instrument for measuring maternal functioning in both clinical and research settings. It was designed in 2010 by Barkin et al. based on the views of 31 postpartum mothers, which were expressed in focus group discussions. It has been argued that, in addition to accessing information about the meanings of desirable and undesirable conditions from individual perspectives, this method makes it possible to get acquainted with the language of the target population [18]. This instrument consists of 20 items that cover all aspects of maternal functioning, including self-care [11], infant care, mother-child interaction, family management, psychological well-being, adjustment, and social support [6, 18–20].

Given the importance of evaluating maternal functioning and the lack of adequate valid instruments in Iran for this purpose, the present study was aimed at translating the tool and conducting a psychometric assessment of the BIMF for Iranian women. The aim of the present study was to adapt the BIMF to the Iranian culture and determine its psychometric properties.

## Methods

### Study’s participants

Those eligible to participate were primiparous women with a vaginal delivery, single pregnancy, self-declared physical health, absence of stressful incidents such as divorce, death of next of kin, and diagnosis of a terminal or refractory diseases for a family member in the past three months, and a lack of a history of depression or any other mental disorder during the pre-pregnancy, prepartum, and postpartum periods (as declared by the participant). Mothers of infants who were hospitalized or had major neonatal anomalies were excluded.

### Sample size

In this study, the sample size required to determine the construct validity of the instrument was considered 10 participants per item [21]. Given the number of questionnaire items (*n* = 20), a sample size of 200 was required. However, the total desired sample size was increased to 530, considering the cluster sampling method (taking into account the design effect equal with 2.5) and an attrition rate of 5%.

### Measurement of functioning: the Barkin index of maternal functioning

The BIMF was used to assess maternal functioning. The BIMF is of comprised 20 items with response options on a 6-point Likert scale. Maternal needs (questions 2, 6, 7, 8, 9, 11, and 13) and maternal competency (questions 1, 3, 4, 5, 10, 12, 14, 15, 16, 17, 18, 19, and 20) were the two underlying factors which emerged in a psychometric evaluation by Barkin et al. [6]. In order to complete the BIMF, Mothers are asked to select the response that best reflects their experience over the past two weeks. The overall maternal functioning score (after reverse-scoring Items 16 and 18) ranges from 0 to 120, with higher scores representing better functioning. In the original study, the Cronbach’s alpha for the BIMF was reported as acceptable (α = 0.87) [6, 18].

### Translation process

Written permission was first obtained from the instrument developer (Dr. Jennifer L. Barkin) for adaptation with the Iranian culture. The original version of the instrument was translated from English into Persian by a native English speaker who was also fluent in Persian. The translated version was reviewed by the research team. The previous version was then translated from Persian into English. At this stage, the translation was done by two translators fluent in both languages who were not involved in the previous stage. The translated version was subsequently reviewed by two individuals fluent in both languages and familiar with the terminology, who prepared the final version.

### Sampling method

Sampling was performed using the cluster method. About one-third of all healthcare centers in Tabriz, Iran, were first selected randomly using the website, www.random.org. Subsequently, the list of mothers during their sixth to tenth postpartum week was prepared based on the medical records at each center. Eligible individuals were randomly selected from the list and invited to participate in the research. The study objectives were then fully explained to mothers via in-person meetings, and the questionnaires were filled out by the participants. It should be noted that mothers were given necessary explanation regarding the study and information regarding confidentiality. Informed written consents were also obtained.

### Data collection

Data collection instruments consisted of a socio-demographic questionnaire and the BIMF, which were completed by the participants from the sixth postpartum week up to the tenth. The socio-demographic questionnaire employed in this study included items regarding maternal age, education, occupation, income status, and unwanted pregnancy. The question about income has been designed as qualitative and the participants’ response was based on their perception of sufficiency of income for household expenses.

### Face and content validity of the Barkin index of maternal functioning

In order to determine the face validity, eight experts in midwifery, reproductive health, and psychiatric nursing were asked to evaluate all items in terms of simplicity and transparency. Based on the answers, the impact of each item was then calculated using the following formula from the score 1 (completely difficult or non-transparent) to 4 (completely simple and transparent) on the Likert scale [Impact = significance (the mean value of the answers to the item) ∗ frequency (number of score 4 s selected)]. Values less than 1.5 resulted in the removal of the item [22].

Content validity was determined quantitatively and qualitatively. For the qualitative portion, the experts who participated in the assessment of face validity were asked to review the translation of each item in terms of grammar, use of proper vocabulary, and proper placement of phrases, and offer their critical comments and revisions. Content validity index (CVI) and content validity ratio (CVR) were used for the quantitative method. CVI was determined by assessing items in terms of relevance, transparency, and simplicity based on a 4-point Likert scale. Scores greater than 0.79 were considered acceptable. In order to determine CVR, experts were asked to examine each item in terms of necessity based on a 4-point Likert scale. The minimum acceptable CVR was considered higher than 0.62 [23, 24].

### Construct validity

Construct validity was assessed through exploratory and confirmatory factor analyses. Exploratory factor analysis was conducted using the Kaiser-Meyer-Olkin (KMO) measure of sampling adequacy and oblimin rotation. Factors were extracted using principal component analysis with varimax rotation, and the number of factors was determined using a scree plot of eigenvalues. An eigenvalue is the amount that determines the variance explained by a factor throughout a dataset. Therefore, the greater the eigenvalue of a factor, the greater its explanatory power of the variance.

The factor analysis method examines the internal relationship between variables and is used to extract categories of items that are most strongly related to each other. In this analysis, items with factor loadings of less than 0.3 were considered candidates for removal. Items with factor loadings of 0.3–0.5 were kept in or removed from the instrument at the research team’s discretion. After extracting factors and expressions therein, their consistency with the dimensions of the original questionnaire was examined [25].

In order to assess the structure of extracted factors from exploratory factor analysis, the model was evaluated using confirmatory factor analysis. Goodness of Fit Index (GFI) was used to assess the exploratory model fit. In order to verify the model, the following indices were determined as follows: Root Mean Square Error of Approximation (RMSEA) < 0.08, the Standardized Root Mean Square Error of Approximation (SRMSEA) < 0.08, Comparative Fit Index (CFI) ≥ 0.90, Tucker-Lewis Index (TLI) ≥ 0.95, and normed chi-square (x^2^ / df) < 5.0 [25, 26].

### Discriminant validity

The discriminant validity and differences between the known maternal age and spousal/family/friend support groups was determined using the known-groups method and Pearson’s correlation coefficient. Studies suggest that younger mothers who receive support enjoy a better maternal functioning [6, 10].

### Reliability

Reliability of the questionnaire was determined using the internal consistency and test-retest reliability methods. Internal consistency and test-retest reliability were examined by calculating the Cronbach’s alpha and the ICC, respectively. Test-retest reliability was evaluated among the mothers who completed the questionnaire twice within a period of two weeks.

## Results

### Participants’ profile

A total of 530 mothers entered the study between August and January of 2018. The mean participant age was 27 years old; the majority of the participating mothers (95.1%) were stay-at- home mothers. Other characteristics are given in Table 1.

**Table 1:** Characteristics of the study participants (*n* = 530)

Characteristics	N (%)
Age (years)	27.0 (5.4)
Education	
High school or below	231 (43.5)
Diploma	195 (36.8)
College	104 (19.6)
Job	
Housewife	504 (95.1)
Employee	26 (4.9)
Income	
Not at all sufficient	80 (15.1)
Relatively sufficient	393 (74.2)
Completely sufficient	57 (10.8)
Unwanted sex of baby	20 (3.8)
Unwanted pregnancy	127 (24.0)
Gestational age at birth (weeks)	38.5 (2.1)

### Face and content validity

The study of face validity indicated that all items had been described as simple and transparent and received a minimum score of 1.5. The study of content validity revealed that all items achieved the minimum acceptable values of CVI and CVR (Table 2).

**Table 2:** The impact score, CVI, and CVR for Barkin Index of Maternal Functioning (BIMF)

BMFI	Impact score	CVI^a^	CVR^b^
	*n* = 8 Experts		
BMFI1	3.04	0.87	0.87
BMFI2	4	1	1
BMFI3	4	1	1
BMFI4	3.04	0.79	0.87
BMFI5	4	1	1
BMFI6	4	1	1
BMFI7	4	1	1
BMFI8	3.04	0.91	1
BMFI9	4	1	1
BMFI10	4	0.91	0.87
BMFI11	4	0.91	1
BMFI12	4	1	1
BMFI13	4	1	1
BMFI14	4	1	1
BMFI15	4	0.79	0.75
BMFI16	4	1	1
BMFI17	4	1	1
BMFI18	3.04	0.95	1
BMFI19	4	1	1
BMFI20	4	1	1

^a^*CVI* Content Validity Index ^b^*CVR* Content Validity Ratio

### Construct validity

Exploratory factor analysis was performed on 20 items through the principal component analysis method. The KMO value was calculated as 0.872. Bartlett’s test achieved a value of 5853.49 at a significant level of less than 0.001, justifying the implementation of factor analysis on the sample based on the correlation matrix.

The number of factors was determined using a scree plot of eigenvalues. Results demonstrated that the highest percentage of the total variance (44.2%) was explained by the first two factors. The remaining of the total variance (12.1%) was explained by the three succeeding factors. Accordingly, two factors with high eigenvalues were identified by this method that accounted for 44.2% of the total variance. Using the scree plot method, two factors were located on the first descending slope. Therefore, using this method, two factors were confirmed. In this study, Item 15 with a factor loading of less than 0.3 and Item 18 with a factor loading of 0.3–0.5 were removed. In addition, Item 16 was not included under any of the factors, hence removed from the Persian version. Finally, the Persian version of the questionnaire was verified with 17 items and two factors, i.e. maternal needs (items 6, 7, and 8) and maternal competency (items 1, 2, 3, 4, 5, 9, 10, 11, 12, 13, 14, 17, 19, and 20) (Table 3).

**Table 3:** Factor loadings of the Barkin Index of Maternal Functioning (BIMF) (*n* = 530)

Items	Factor 1	Factor 2
BMFI1	0.525	
BMFI2	0.585	
BMFI3	0.454	
BMFI4	0.750	
BMFI5	0.688	
BMFI6		0.936
BMFI7		0.936
BMFI8		0.413
BMFI9	0.610	
BMFI10	0.762	
BMFI11	0.504	
BMFI12	0.739	
BMFI13	0.537	
BMFI14	0.775	
BMFI15		0.269
BMFI16		
BMFI17	0.723	
BMFI18	0.329	
BMFI19	0.779	
BMFI20	0.654	
% Variance Explained	33.2	10.9

**Fig. 1:**
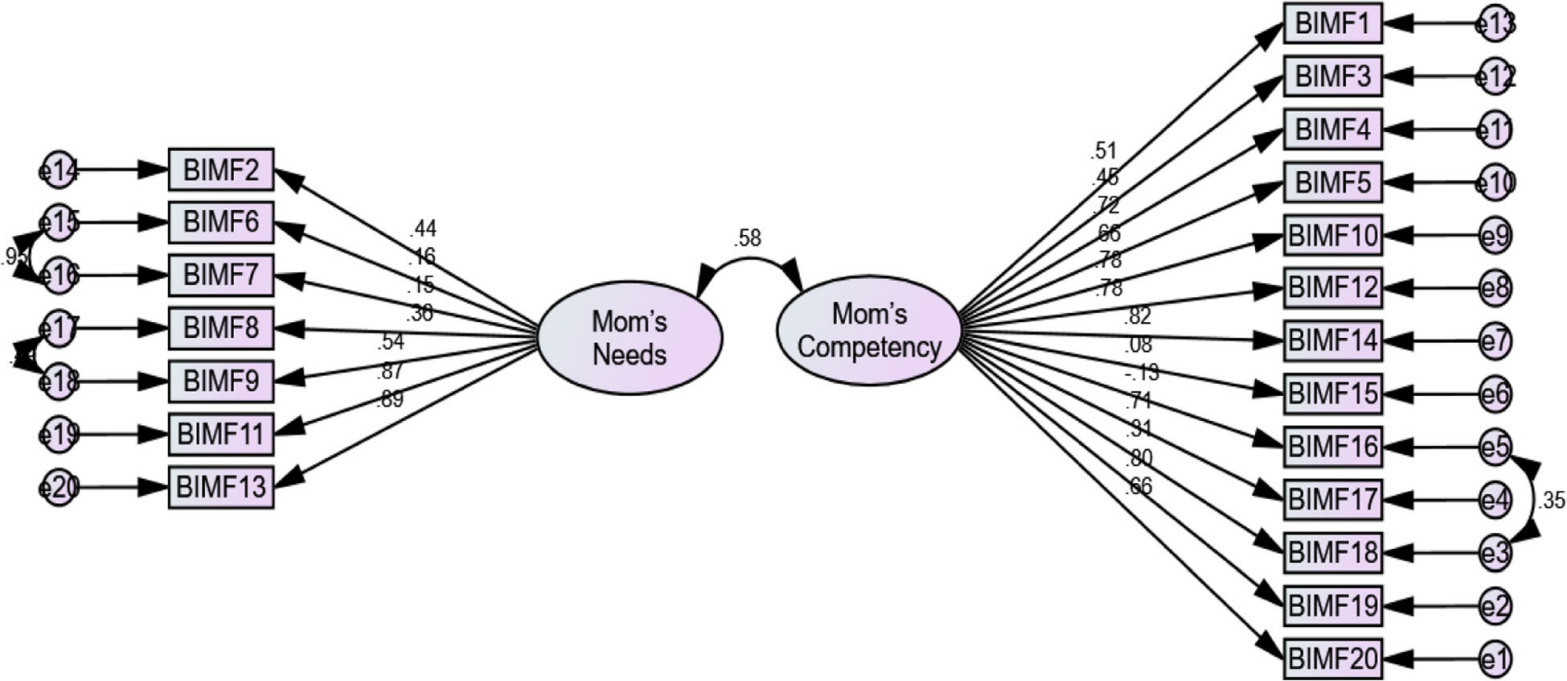
CFA factor loading for Barkin Index of Maternal Functioning (BIMF)

Given the values of indices in Table 4, the *x*^*2*^*/df* ratio was smaller than 5, and the RMSEA and RMR values were smaller than 0.08 and 0.1, respectively, verifying the model validity. Moreover, the GFI and AGFI were greater than 0.9, demonstrating the verifiability of their factor structure and the acceptable model fit (Table 4). Given the relative fit of the confirmatory factor model and the significant item-scale relationship, the results of the exploratory factor model were supported by confirmatory patterns, and the construct validity of the scale was verified (Fig. 1).

**Table 4:** Confirmatory factor analyses fit Index of the Barkin Index of Maternal Functioning (BIMF) (*n* = 530)

Fit Indices	Fit
$$ \raisebox{1ex}{${x}^2$}\!\left/ \!\raisebox{-1ex}{$ df$}\right. $$	4.90
RMSEA (90%CI)	0.07 (0.073 to 0.08)
GFI	0.94
AGFI	0.90
NFI	0.92
IFI	0.95
TLI	0.93
CFI	0.95

$$ \raisebox{1ex}{${x}^2$}\!\left/ \!\raisebox{-1ex}{$ df$}\right. $$: Normed chi-square; *RMSEA* Root Mean Square Error of Approximation, *GFI* Goodness of Fit Index, *AGFI* Adjusted Goodness of Fit Index, *NFI* Normed Fit Index, *IFI* Incremental Fit Index, *TLI* Tucker- Lewis Index, *CFI* Comparative Fit Index

### Discriminant validity

Maternal age had a significant inverse correlation with the overall maternal functioning score (r = − 0.18, *p* < 0.001). In addition, maternal age had a significant inverse correlation with the sub-scales of maternal needs (r = − 0.19, *p* < 0.001) and maternal competency (r = − 0.11, *p* = 0.008). Gestational age did not have a significant correlation with the overall maternal functioning score and its sub-scales (*P* > 0.05). A significant difference in the maternal needs (*p* < 0.001) and the overall maternal functioning score (*p* < 0.001) was observed between mothers who had been supported and those who had not received any support (Table 5).

**Table 5:** Discriminante validity for the Barkin Index of Maternal Functioning (BIMF) and it’s sub scales (*n* = 530)

Variables	Mom’s needs	Mom’s competency	Total
Mother’s age			
r	−0.19	−0.11	−0.18
*P*-value*	< 0.001	0.008	< 0.001
Receiving support			
Yes^&^	34.3 (6.6)	63.5 (6.9)	97.9 (11.8)
No^&^	28.1 (6.5)	62.3 (7.0)	90.5 (11.1)
*P*-value**	< 0.001	0.054	< 0.001

^*^Pearson correlation ^&^Mean (SD) **Independent T-test

### Reliability

Cronbach’s alpha coefficient of the questionnaire items was calculated from 0.78 (maternal competency) to 0.86 (maternal needs). Cronbach’s alpha coefficient of the overall 20-item version was calculated as 0.88, indicating the acceptable internal consistency of the questionnaire. In the test-retest method, the ICC (95% Confidence Interval) of the total questionnaire, and the constructs of maternal needs and maternal competency were calculated as 0.85 (0.64 to 0.94), 0.89 (0.59 to 0.93), and 0.88 (0.71 to 0.95), respectively (Table 6).

**Table 6:** Mean (SD), Cronbach’s alpha, and ICC for the Barkin Index of Maternal Functioning (BIMF) (*n* = 530)

BMFI	Mean (SD^a^)	Possible range	Obtainable range	Cronbach’s alpha	ICC (95% CI)^b^
Mom’s needs	32.0 (7.2)	0–42	7–42	0.86	0.89 (0.59 to 0.93)
Mom’s competency	63.1 (6.9)	0–78	27–78	0.78	0.88 (0.71 to 0.95)
Total	95.2 (12.1)	0–120	39–119	0.88	0.85 (0.64 to 0.94)

^a^*SD* Standard Deviation ^b^*ICC (95% CI)* Intarclass Correlation Coefficient (95% Confidence Interval)

## Discussion

The present study was conducted in order to assess the psychometric properties of the BIMF in a sample of Iranian mothers. The results showed that the Persian version of the BIMF is a valid and reliable instrument for assessing maternal functioning among Iranian mothers. The face, content and construct validity, internal consistency, and test-retest reliability of the instrument were all confirmed.

Based on exploratory analysis, Items 15 and 18 were removed from the Persian version on account of their low factor loadings. In addition, Item 16 was not included under any of the factors and was therefore removed. Items 16 and 18 had also been removed in the psychometric assessment of the Turkish version of the instrument due to their low factor loadings. Moreover, Item 15 was reported to have been inappropriately labeled under one of the factors. The findings of the psychometric assessment of the Turkish version are consistent with the Persian version [27]. The culture of Iran is similar to Turkey’s and thus, the consistency is justified.

Barkin et al. (2014) [6] evaluated the psychometric properties of the original version, in which they also excluded Items 16 and 18 from their analysis due to inadequate factor loadings. Barkin et al. eventually introduced an 18-item version of the instrument as well, though they recommend the 20-item version as it includes questions on anxiety and worry, which are important considerations in the postpartum period. Items 16 and 18 are the only items that address anxiety, worry, and mother-child interaction and they are valuable for practical purposes. For example, if an organization assessed maternal functioning using a shortened version of the BIMF, but did not screen for depression simultaneously, there would be no gauge of maternal anxiety, which is highly prevalent in the postpartum period [28]. Therefore, the Persian version of the BIMF can be administered in its 17-item version or in its original 20-item version.

Two factors were extracted from the Persian version: maternal needs (items 6, 7, and 8) and maternal competency (items 1, 2, 3, 4, 5, 9, 10, 11, 12, 13, 14, 17, 19, and 20). The number of factors extracted from the Turkish version is not consistent with those from the Persian version [27]. In the Turkish version, the five factors are named “self-care”, “child care”, “maternal psychology”, “maternal attachment”, “maternal management”, “social support”, and “compatibility with the maternal role” [27]. However, the number of factors extracted from this study is consistent with those from the Barkin et al.’s (2014) study. In their study of the psychometric properties of the instrument, they also discovered only two latent factors “maternal needs” and “maternal competency” [6].

The internal consistency was 0.88 for the Persian version and ranged from 0.78 (maternal competency) to 0.86 (maternal needs) for its dimensions. In the original (English) version [6], the Cronbach’s alpha coefficients of the 20-item version, and the dimensions of maternal needs and maternal competency were 0.87, 0.77, and 0.88, respectively - in line with the results of the internal consistency of the Persian version. However, the Cronbach’s alpha of the Turkish version [27] was reported as 0.73, which was lower than that of the Persian version. Such a distinction can be attributed to the greater number of items in the factors of the Persian and English versions, as items are scattered around five factors in the Turkish version instead of two. The ICC of “maternal needs” and “maternal competency” in the Persian version was 0.89 and 0.88, respectively, which was consistent with the English version (0.80 and 0.88).

In this study, the total BIMF score and its sub-scale’s scores had a significant inverse correlation with maternal age. In other words, older mothers had a weaker maternal functioning than younger mothers. Barkin et al. [19] also found a significant inverse correlation between maternal age and maternal competency, which is relatively consistent with the findings of this study. In addition, a significant difference in the maternal needs and the overall maternal functioning score was observed between mothers who had been supported and those who had not received any support. In a study by Giallo et al. [10] women who had been supported by home visits during the postpartum period were more capable of adapting to anxiety and depression and exhibited more favorable maternal behavior than the control group. Another study also reported that women who are supported during the postpartum period through pre-natal and postnatal training programs, home visits and telephone counseling, feel more self-sufficient and competent in self-care and neonatal care and have a better maternal functioning [29].

### Strengths and limitations

The large sample size and the use of random cluster sampling are strengths and enhance the generalizability of this study to Iranian mothers outside of the study population. Non-inclusion of mothers with cesarean and multiparous delivery is a limitation of this study, as the psychometric assessment of the instrument is not applicable to all of these groups. However, the fact that our results are largely in agreement with the Barkin et al. (2014) psychometric analysis -which did not exclude women who had a cesarean section– is reassuring as to the generalizability. Failure to account for mothers affected by intimate partner violence -a proven stressor– could be considered an omission. However, none of the related studies thus far have included intimate partner violence as an explanatory variable.

## Conclusion

The findings of this study revealed that the Persian version of the BIMF is a valid and reliable instrument for assessing postpartum maternal functioning in Iranian women. This instrument can be used by healthcare providers such as doctors, midwives, and nurses to screen and examine the compatibility of mothers with the maternal role in the postpartum period.

## Data Availability

Not applicable.

## Abbreviations

AGFI: Adjusted Goodness of Fit Index
BIMF: Barkin Index of Maternal Functioning
CFI: Comparative Fit Index
CVI: Content validity index
CVR: Content Validity Ratio
GFI: Goodness of Fit Index
ICC: Intra-class Correlation Coefficient
IFI: Incremental Fit Index
IFSAC: Inventory of Functional Status After Childbirth
KMO: Kaiser-Meyer-Olkin
NFI: Normed Fit Index
RMR: Root Mean Square Residual
RMSEA: Root Mean Square Error of Approximation
RMSEA: Root Mean Square Error of Approximation
TLI: Tucker- Lewis Index

## References

[1] Mercer RT (2006). Nursing support of the process of becoming a mother. J Obstet Gynecol Neonatal NSurs.

[2] Don BP, Chong A, Biehle SN, Gordon A, Mickelson KD (2014). Anxiety across the transition to parenthood: change trajectories among low-risk parents. Anxiety Stress Coping.

[3] Meleis AI (2010). Transitions theory: middle range and situation specific theories in nursing research and practice: springer publishing company.

[4] Negron R, Martin A, Almog M, Balbierz A, Howell EA (2013). Social support during the postpartum period: mothers' views on needs, expectations, and mobilization of support. Matern Child Health J.

[5] Nelson AM (2003). Transition to motherhood. J Obstet Gynecol Neonatal Nurs.

[6] Barkin JL, Wisner KL, Wisniewski SR (2014). The psychometric properties of the Barkin index of maternal functioning. J Obstet Gynecol Neonatal Nurs.

[7] Smith-Hanrahan C, Deblois D (1995). Postpartum early discharge: impact on maternal fatigue and functional ability. Clin Nurs Res.

[8] McVeigh CA (2000). Anxiety and functional status after childbirth. Aust Coll Midwives Inc J.

[9] Ngai FW, Wai-Chi Chan S, Ip WY (2010). Predictors and correlates of maternal role competence and satisfaction. Nurs Res.

[10] Giallo R, Cooklin A, Dunning M, Seymour M (2014). The efficacy of an intervention for the management of postpartum fatigue. J Obstet Gynecol Neonatal Nurs.

[11] Barkin JL, Wisner KL (2013). The role of maternal self-care in new motherhood. Midwifery..

[12] Barkin JL, Wisner KL, Bromberger JT, Beach SR, Wisniewski SR (2010). Assessment of functioning in new mothers. J Women’s Health (Larchmt).

[13] Barkin JL, Beals L, Bridges CC, Ezeamama A, Serati M, Buoli M, ... & Bloch JR. Maternal Functioning and Depression Scores Improve Significantly With Participation in Visiting Moms® Program. J Am Psychiatr Nurses Assoc. 2019;1–10. 10.1177/1078390319877444 31561726

[14] Fawcett J, Tulman L, Myers ST (1988). Development of the inventory of functional status after childbirth. J Nurse Midwifery.

[15] McVeigh C (1998). Functional status after childbirth in an Australian sample. J Obstet Gynecol Neonatal Nurs.

[16] Aktan NM (2007). Functional status after childbirth: a review of the literature. Clin Nurs Res.

[17] McVeigh C, Chaboyer W (2002). Reliability and validity of the inventory of functional status after childbirth when used in an Australian population. Nurs Health Sci.

[18] Barkin JL, Wisner KL, Bromberger JT, Beach SR, Terry MA, Wisniewski SR (2010). Development of the Barkin index of maternal functioning. J Women's Health (Larchmt).

[19] Barkin JL, Willis GB, Hawkins KC, Stanfill-Thomas T, Beals L, Bloch JR (2017). Semantic assessment of the Barkin index of maternal functioning in a medically underserved obstetric population. Perspect Psychiatr Care.

[20] Barkin JL, Bloch JR, Hawkins KC, Thomas TS (2014). Barriers to optimal social support in the postpartum period. J Obstet Gynecol Neonatal Nurs.

[21] Nunnally JC, Bernstein IH. Psychometric Theory. New York: Mc Graw-hill; 1994.

[22] Seyf AA. Measurement, test and educational evaluation. 7 ed. Tehran: Douran; 2016.

[23] Hajizadeh E, Asghari M. Statistical methods and analyses in health and biosciences a research methodological approach. Tehran: Jahade Daneshgahi Publications. 2011;395–450.

[24] Lawshe CH (1975). A quantitaitive approach to content validity. Pers Psychol.

[25] Tinsley HE, Brown SD (2000). Handbook of applied multivariate statistics and mathematical modeling: academic press.

[26] Bentler PM, Bonett DG (1980). Significance tests and goodness of fit in the analysis of covariance structures. Psychol Bull.

[27] Aydin R, Kukulu K (2018). Adaptation of the Barkin scale of maternal functioning and examination of the psychometric properties. Health Care Women Int.

[28] Fairbrother N, Janssen P, Antony MM, Tucker E, Young AH (2016). Perinatal anxiety disorder prevalence and incidence. J Affect Disord.

[29] Aydın R, Barkin JL, Kukulu K (2016). Attempts to strengthen maternal functioning in the postpartum period: a literature review. J Hum Sci.

